# Comparative Study of Cowpea Storage Technologies in the Sahel Region of Niger

**DOI:** 10.3390/insects11100689

**Published:** 2020-10-12

**Authors:** Ousmane N. Bakoye, Baoua Ibrahim, Haoua Seyni, Laouali Amadou, Larry L. Murdock, Dieudonne Baributsa

**Affiliations:** 1 Département des Sciences et Techniques de Productions Végétales, Université Dan Dicko Dankoulodo de Maradi, Maradi BP 465, Niger; ousmanebakoy@yahoo.fr (O.N.B.); baoua.ibrahim@gmail.com (B.I.); 2 Département de Chimie, Université Abdou Moumouni de Niamey, Niamey BP 10662, Niger; hseinisabo@yahoo.fr; 3 Institut National de la Recherche Agronomique du Niger (INRAN), Maradi BP 240, Niger; amadoulaouali@gmail.com; 4 Department of Entomology, Purdue University, West Lafayette, IN 47907, USA; murdockl@purdue.edu

**Keywords:** cowpea pest, airtight storage, scaling-up technologies, smallholder farmers, West Africa

## Abstract

**Simple Summary:**

Cowpea farmers in the West Africa lose a significant portion of their crop during storage due to insects (cowpea weevil). To help farmers deal with this issue, we tested several storage technologies including hermetic (SuperGrainbag^TM^, AgroZ^®^ bag, EVAL^TM^, and Purdue Improved Crop Storage-PICS^TM^ bags), an insecticide-treated woven bag (ZeroFly^®^), and a polypropylene (PP) woven bag. After 8 months of storage, we observed that all hermetic bags were effective at maintain the quality of cowpea. No further damage and weight loss was observed in grain stored in hermetic bags; germination decreased modestly, by a maximum of 16%. However, grain stored in ZeroFly^®^ and woven bags continued to deteriorate during the storage period due to continued insect population growth, resulting in weight loss of about 25%. Loss in germination was more serious in cowpea stored in the ZeroFly^®^ (37.0%) and woven bags (28.8%) than in hermetic bags. Farmers and development agencies in the West Africa (particularly in the Sahel) can use and/or recommend these hermetic technologies to greatly reduce cowpea storage losses due to insects.

**Abstract:**

Cowpea stored on smallholders’ farms suffers serious losses to insect pests. A study conducted in Niger compared five postharvest technologies marketed in sub-Saharan Africa to protect stored grain. Naturally-infested cowpea stored for eight months showed adult *Callosobruchus maculatus* (F.) mortality of 97% to 100% in the hermetic bags (PICS^TM^, SuperGrainbag^TM^, AgroZ^®^, EVAL™, and ZeroFly^®^ bags). There was no change in grain damage and weight loss of cowpea stored in hermetic bags. There was, however, a loss of up to 10 to 16% in germination when the grain was stored in hermetic bags. Results observed for grain stored in ZeroFly^®^ bags impregnated with deltamethrin were substantial and similar to those in control woven bags. In both ZeroFly^®^ and woven bags, (1) adult *C. maculatus* population augmented by 35.7% and 78.6%, (2) increased weight losses of 27.3% and 25.2%, and (3) reduced germination of 37.0% and 28.8%, respectively. After opening the bags, abrasions were noted on the liners of hermetic bags, potential damage that could limit their reuse if they only have a single liner. Smallholder farmers in the Sahel can safely store their cowpea in all the hermetic bags tested. However, further research is needed to mitigate insect damage on liners of hermetic bags to improve their performance and reusability.

## 1. Introduction

Cowpea (*Vigna unguiculata* L. Walp) is an ancient crop that originated and was domesticated near Ethiopia [1]. Cowpea, widely cultivated in West Africa, is an important source of protein, and plays a major role in generating income and ensuring food security for many smallholder farmers [2,3,4,5]. In Niger, cowpea represents a quarter of all agricultural grain production with about 2 million tons produced in 2017 [6]. Niger is the second producer of cowpea only after Nigeria; both countries produce about 70% of the world production [7]. Niger, like many other countries in West Africa, has the potential and the need to increase cowpea productivity; however, there are many constraints. Among challenges to increase cowpea productivity and utilization are the availability of quality seed, together with field, postharvest, and market constraints [8]. Insect pests are the major postharvest storage challenge in the Sahel. *Callosobruchus maculatus* (F.) (Coleoptera: Crysomelidae), the most important cowpea storage pest, is responsible for losses of up to 60% or higher during storage [9,10].

Cowpea storage in West and Central Africa has significantly improved over the last decade thanks to the introduction and large-scale dissemination of the Purdue Improved Crop Storage (PICS^TM^) bags [11,12]. PICS^TM^ is a chemical-free hermetic technology that maintains grain quality by stopping insect development and reproduction in the commodity stored inside the bag [13]. After the PICS^TM^ bag is filled with infested grain and closed, insects consume the remaining oxygen inside the container leading to hypoxia and eventual death by desiccation [13]. PICS^TM^ bags are effective at protecting a wide range of cereal and legume crops during storage, including cowpea against *C. maculatus* [13,14,15,16,17]. 

Wide dissemination of PICS^TM^ bags led to the development and commercialization of several alternative hermetic bags as well as other storage technologies in sub-Saharan Africa, especially in East and Southern Africa [18]. In addition to PICS^TM^ bags, other storage technologies that have been tested for maize storage in sub-Saharan Africa include SuperGrainbag^TM^, AgroZ^®^, EVAL™, and ZeroFly^®^ bags [15,19,20,21,22,23,24,25,26,27,28,29]. These studies have provided evidence that hermetic bags are effective at protecting grain during storage, while the ZeroFly^®^ bag has limited success. A few studies have compared several hermetic storage bags for cowpea storage [15,30]. Results suggest that all these hermetic bags are effective at preserving cowpea for more than six months. 

The dissemination of the PICS bags for cowpea storage in West and Central Africa has helped farmers reduce postharvest losses, improve food security, and increase income [31,32]. As of 2014, the PICS^TM^ technology had reached more than 2.5 million farmers in about 31,000 villages in West and Central Africa [12]. Adoption studies have shown that one of the challenges to further increase the use of the technology by farmers is the unavailability of PICS^TM^ bags in rural areas due to the limited private sector distribution networks [12,32,33]. Several development organizations and private sector companies have since become interested in expanding the market for hermetic and other storage solutions in West Africa for cowpea storage. The present research was conducted to assess the performance of existing and recently commercially available storage solutions for cowpea preservation before major efforts are undertaken to scale them up in the Sahel. The results will be useful to cowpea producers and traders, as well as development partners interested in using or promoting postharvest storage solutions in the Sahel. 

## 2. Materials and Methods

The experiments described here were part of an effort to test several hermetic and other storage solutions for grain storage in the Sahel. This study was conducted at the National Agricultural Research Institute of Niger (INRAN) Maradi station (latitude: 13.8460 N, longitude: 07.8080 E) located in the south-central region of Niger. The study was launched on 1 August 2016 during the rainy season and was completed on 30 March 2017 during the dry season (a total of 218 days or about eight months). Tested storage solutions included four hermetic bags (PICS^TM^, SuperGrainbag^TM^, AgroZ^®^, EVAL™) as well as the ZeroFly^®^ bag, a woven bag impregnated with an insecticide (deltamethrin). Conventional polypropylene (PP) woven bags typically used by farmers for grain storage were used as a control. Detailed characteristics of the various bags used in this experiment including brand, composition, thickness, and suppliers are provided in a similar study evaluating the use of hermetic bags for maize storage in Benin [19]. Liners were checked for holes or tears by filling them with air and bags were closed using strings after filling them with cowpea [34]. Naturally-infested cowpea was thoroughly mixed before filling 50 kg in each of the 4 replicates of every treatment (hermetic bag type). Bags filled with cowpea were randomly stored in four blocks, each containing a treatment. After filling, the bags were stored at ambient temperature and relative humidity in a laboratory at INRAN Maradi for the duration of the experiment. 

Cowpea infestation was assessed using twelve (*n* = 12) samples of 500 g each randomly collected per treatment (three samples per replicate) at the beginning and the end of the trial. Each 500 g was sieved to separate and count live adults of cowpea bruchids. Three sub-samples of 100 grains were randomly collected from each of the three 500 g samples, resulting in 900 grains per replicate (*n* = 36 per treatment). These 100-grain sub-samples were used to estimate grain with eggs, grain damage, and grain dry weight. We determined insect-damaged grain [35] and the weight losses [36] using the formulas below:

$$\%\;Insect\;-damaged\;grain=\;\frac{\left(Numberofdamagedgrains\right)}{Total\;grain\;count}\;\times 100$$

$$\%\;weight\;loss=\;\frac{DWo\;-DWt}{DWo}\;\times 100$$

where *DWo* is dry weight at the beginning, and *DWt* is dry weight at the end. 

To assess germination, we used two sub-samples of 25 grains that were randomly taken from each of the three 500 g samples (*n* = 24 per treatment), resulting in 150 seeds per replicate. Percentage grain germination was calculated as shown below:

$$\%\;Germination=\;\frac{Number\;of\;germinated\;grains}{Total\;number\;of\;grains\;tested}\times 100$$

Grain moisture content was assessed at the start and the end of the experiment using the Dickey John mini GAC (Dickey-John, Auburn, IL, USA). The oxygen level in each bag were recorded several times (24 h and 8 d after closing the bags, and every month thereafter) for the first four months using a non-invasive method—OxySense 5250i (Devens, MA, USA). The OxySense 5250i relies on a light sensitive oxygen sensor, OxyDot^®^, that fluoresces under ultraviolet light. The OxySense 5250i reads and interprets this fluorescence, and displays an oxygen concentration level in a given volume. The OxyDots^®^ are first attached to the inside of the container prior to sealing. Measurements are taken from outside the container over the OxyDot^®^ by holding the fiber-optic reader pen attached to the OxySense 5250i. Temperature and relative humidity were recorded every hour during the trial using data loggers (EL-USB-2 model, Lascar; Erie, PA, USA) placed in one bag of each treatment. Data presented in the plots are daily averages of temperature and relative humidity. At the end of the trials, liners of hermetic bags were inspected to assess insect abrasions and perforations (holes).

We used Microsoft Excel (Microsoft Office, Redmond, WA, U.S; version 2013) to estimate means and standard errors of means. SPSS 24.0 (IBM Corporation, Armonk, NY, USA) was used to conduct statistical analysis. The analysis of variance (ANOVA) followed by least significant difference (LSD) was used to compare means of oxygen content, germination, weight loss, grain with holes, grains with eggs, and liner abrasions and perforations.

## 3. Results

Oxygen levels measured during a four-month period showed significant differences among treatments as soon as one day after closing the bags (F = 50.04; df = 5/18; *p* < 0.01); the same was the case four months later (F = 10.16; df = 5/18; *p* < 0.01) (Table 1). Oxygen levels in the bags dropped steeply, reaching below 1.5% (*v/v*) in all hermetic bags 8 days after closing the bags, but these levels significantly increased from the second to the fourth month. Oxygen in both the ZeroFly^®^ and woven bag treatments remained at the ambient levels during the four months. 

**Figure 1:**
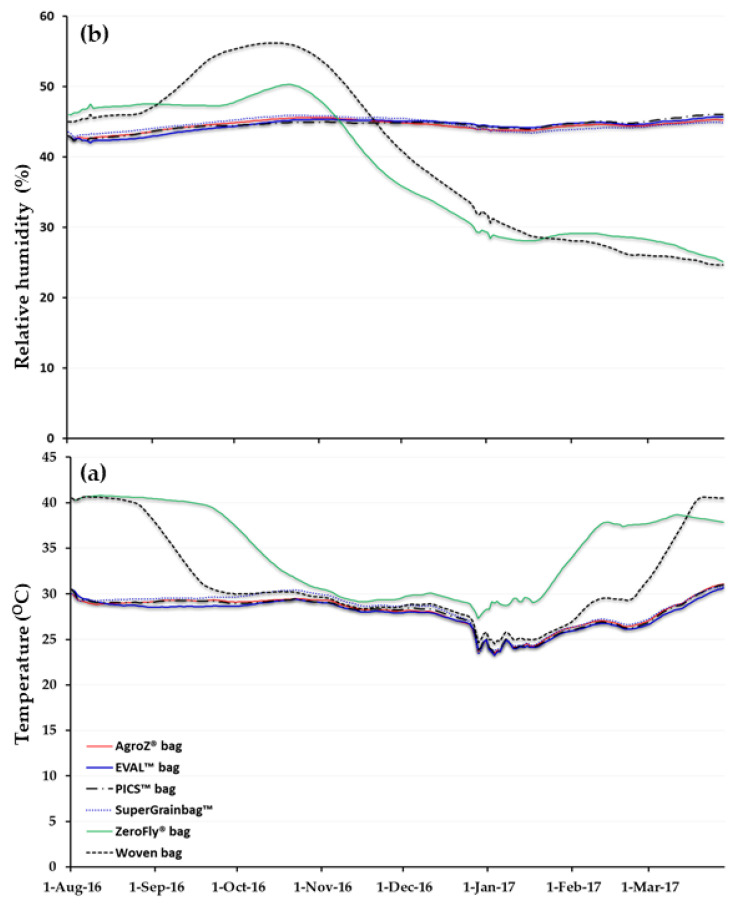
(**a**) daily average temperatures and (**b**) relative humidities in six types of hermetic and non-hermetic bags filled with naturally-infested cowpea stored for eight months in Maradi, Niger.

Average daily temperatures and relative humidities varied among the treatments during the 8-month storage period (Figure 1). Average temperatures among all of the hermetic bags exhibited the same trends (Figure 1a). Average relative humidities varied minimally (less than ±2.5%) among the various hermetic treatments (Figure 1b). By contrast, ZeroFly^®^ and woven bags exhibited average temperatures that decreased and then slowly increased toward the end of the storage. However, relative humidity increased in the first three months and then consistently decreased until the end of the experiment. 

The cowpea used in this trial had an initial infestation of 22.35 *C. maculatus* adults per 500 g (Table 2). After eight months of storage, there was a decrease in the numbers of adult weevils of between 97.05% and 100% in all hermetic bag treatments. However, in the ZeroFly^®^ and woven bag treatments, there was an increase of 35.70% and 78.59% in the adult insect population, respectively. Grain stored in each of the hermetic bags maintained the same level of insect damages (holes) throughout the storage period as was present initially (Table 2). By contrast, there was an increase in damage of 61.47% and 59.36% of grain stored in ZeroFly^®^ and woven bags, respectively. The proportions of seeds carrying *C. maculatus* eggs were comparable between the treatments within the airtight bags, whereas, in the ZeroFly^®^ and control woven bag treatments, there was an increase of 63.80% and 65.80% in grain with eggs, respectively (Table 2).

The weights of 100 grains from hermetic bag treatments after eight months of storage were comparable to those observed at the beginning of the experiment (Table 3). However, in the ZeroFly^®^ and control woven bag treatments, there was additional weight loss of 27.26% and 25.21%, respectively. There was no significant change in moisture content of cowpea from the beginning (8.1%) to the end of the experiment (8.7%); hence, we did not adjust the DW while estimating weight loss. There was a decrease in germination rates of about 10–16% of grain stored in hermetic bags, while it decreased by 37.0% in ZeroFly^®^ bags and by 28.75% in control woven bags (Table 3). 

At the end of the experiment, we observed abrasions and perforations on the liners of each type of hermetic bags, but there was not significant difference among the treatments (Table 4). For PICS^TM^ bags, perforations were only observed on the first (most inner) liner, but not on the second (middle) liner.

## 4. Discussion

The increase in insect populations and damages to grain stored in ZeroFly^®^ and control woven bags observed here corroborates findings of previous studies in which maize was stored in several different countries in sub-Saharan Africa [19,25,27,28,29]. The present study further confirmed that *C. maculatus* is the only insect species that attacks cowpea during storage in West Africa. In Zimbabwe, *Callosobruchus rhodesianus* (Pic.) was identified as the major insect pests of cowpea during storage [30]. The high survival rates of adult insects in grain stored in ZeroFly^®^ and control woven bags for eight months may be explained by *C. maculatus’* ability to withstand harsh environmental conditions such as high temperatures and low relative humidities [37], conditions similar to those observed in this study. *C. maculatus*, the major pest of cowpea, an indigenous crop in the dry Africa savannah, has adapted to these extreme environmental conditions. By contrast, insect populations of infested maize stored in both ZeroFly^®^ and control woven bags in northern Benin decreased as temperatures increased and relative humidity decreased [19]. ZeroFly^®^ storage bags do not adequately control *C. maculatus* because deltamethrin impregnated into the weaves of the woven bag does not affect eggs, larvae, and pupae as well as adults that do not come into direct contact with the bag wall. 

Our study demonstrates that hermetic bags can significantly reduce insect populations and damage to infested grain stored for several months. This is consistent with several studies comparing hermetic bags for cowpea preservation in Niger (PICS^TM^ and SuperGrainbag^TM^ bags) and in Zimbabwe (SuperGrainbag^TM^, PICS^TM^, AgroZ^®^, AgroZ^®^Plus, and ZeroFly^®^ hermetic bags) that showed complete suppression of adult cowpea bruchids, minimal additional grain damage, and a reduction in grain carrying eggs [15,30]. Most of these studies, like the current one, point to the efficacy of hypoxic environment in suppressing or minimizing the development of and damage by insects in infested grain stored in hermetic bags. Hypoxia with oxygen levels below 5% that prevailed for at least a fortnight during the first month of storage in this and other studies explains the effectiveness of hermetic bags. Such conditions are unfavorable to bruchid activities [38] and may explain the high mortality rates, which range between 97% to 100%. These mortality rates are similar to those reported in Niger (92–100%) during cowpea storage in PICS^TM^ and SuperGrainbag^TM^ [15]. The rise in oxygen levels after a sharp decrease due to insect activities has been observed in previous studies [14,39]. This rise is due to oxygen leaks into the bag from outside of the bags as the liners are not completely airtight. 

Hermetic bags are effective at preserving grain quality by preventing further development of insect pests and hence grain weigh loss [13,14]. Despite differences in the composition of the various brands of hermetic bags (single versus double liners, and single versus multilayered-liners), the trend of oxygen depletion was the same during the first four months of the experiment. The quick drop in oxygen in less than two weeks after the launch of the experiment probably explains the negligible weigh loss (less than 3.5%) in these hermetic bags. Previous studies have shown similar weight losses during cowpea storage in Niger and other countries [9,14]. The large weight loss in control woven bags of 27.26% is comparable to the losses of 16.7% to 40% observed after 4.5 to 5 months of cowpea stored in Niger [14,40]. Similar to control woven bags, weight losses in grain stored in ZeroFly^®^ storage bags increased over the initial value after eight months of storage, suggesting that deltamethrin did not substantially reduce or prevent the development of insects during storage. Our results are corroborated by findings from other studies on maize in several regions of sub-Saharan Africa. These studies showed weigh losses ranging from 3% to 35% [19,20,22,25,27,28,29]. Our results contradict laboratory studies conducted in both Ghana and Nigeria that claimed 100% insect mortality and no weight loss of cowpea stored in 5 and 10 kg ZeroFly^®^ storage bags for 21 to 25 days [41]. These results are likely explained by (1) the extremely high surface-to-volume ratio in the small (5 and 10 kg) ZeroFly^®^ storage bags used in the Vestergaard experiments and (2) the extremely short duration of the experiments. *C. maculatus* may have died due to their short lifespan as adults may live only for about 10 days [42,43].

ZeroFly^®^ storage bags are better suited for preventing infestation from outside of the bags than for controlling insects in grain stored inside the bags. Pre-fumigated maize stored in ZeroFly^®^ storage bags had insect-damaged grain of up 10% after 6 months [44]. When using ZeroFly^®^ storage bags, it is recommended that grain first be disinfested (by fumigation or solarization) before storage or fumigated in case an infestation occurs during storage [41,45]. Most smallholder farmers store infested grains and would have challenges disinfesting it. Hence, it would be better for smallholder farmers to use the new ZeroFly^®^ hermetic storage bags because they have shown to be effective at preserving infested cowpea during storage [30]. ZeroFly^®^ hermetic storage bags are ZeroFly^®^ storage bags fitted with a liner inside, to create an airtight environment [46]. Storing grain in hermetic bags has shown to maintain or minimally reduce its germination [40]. Significant losses of germination in grain stored in ZeroFly^®^ storage bags and woven bags described here are likely due to grain damage due to insects [13,28,47]. 

A comparable number of abrasions and perforations observed on the single liners of AgroZ^®^, SuperGrainbag^TM^, EVAL™ bags, and the inner liner of the PICS^TM^ bag have been reported by other studies [15,19,26,29]. A recent study from Zimbabwe reported similar findings and also noted insects making holes on the single liner of the ZeroFly^®^ hermetic bag [30]. An increase in the number of holes on liners can eventually decrease the efficacy of these hermetic bags [48]. The lack of or severely limited damage to the second liner of the PICS^TM^ bag may help increase its effectiveness and extend its useful life [16,18,19]. The composition of the liners of these different hermetic bag brands (single layer or multilayers and single liner or two liners) appears to have a minimal effect on their oxygen permeability and insect damage. A study conducted in Niger suggests that perforations by cowpea bruchids are made during the emergence of adults when the exit holes are against the liners [15]. There is a need to understand better how insects are able to make holes in the liners regardless of bag properties. This would help improve the performance and reusability of these hermetic bags. Beyond insect damage on the liners, potential exposure of these hermetic bags to the Sahelian extreme environmental conditions (e.g., stored outside for a limited time) would further reduce their longevity and reusability. A study conducted in Niger showed that a PICS bag stored outside and exposed to the sunlight cannot be reused and would only maintain cowpea quality for about 4.5 months [40].

## 5. Conclusions

Our results demonstrate that several brands of hermetic bags currently marketed in sub-Saharan Africa, including AgroZ^®^, SuperGrainbag^TM^, EVAL™, and PICS^TM^ bags are suitable and effective for cowpea storage on smallholder farms in the Sahel. Scaling-up more hermetic bag brands in the Sahel would help improve the availability of these storage solutions and provide more options to smallholder farmers. 

## Figures and Tables

**Table 1:** Change in oxygen levels during the first four months in hermetic and non-hermetic bags filled with naturally infested cowpea stored for eight months in Maradi, Niger.

	Oxygen Concentration (%; Mean ± Standard Error of the Mean)					
Treatments	2/Aug/2016	10/Aug/2016	2/Sep/2016	2/Oct/2016	2/Nov/2016	2/Dec/2016
PICS^TM^ bag	14.13 ± 0.39 b	1.16 ± 0.21b	5.04 ± 0.75b	15.55 ± 0.55b	18.34 ± 0.13b	19.33 ± 0.15a
AgroZ^®^ bag	15.33 ± 0.33b	0.94 ± 0.13b	5.02 ± 0.94b	14.95 ± 0.82b	18.08 ± 0.10b	19.22 ± 0.24a
SuperGrainbag^TM^	14.59 ± 0.66b	1.24 ± 0.17b	3.99 ± 0.58b	11.74 ± 0.85c	13.28 ± 1.19d	16.72 ± 0.92b
EVAL^™^ bag	14.63 ± 0.59b	0.96 ± 0.21b	4.08 ± 0.64b	12.54 ± 0.65c	16.36 ± 0.33c	18.93 ± 0.11a
ZeroFly^®^ bag	20.46 ± 0.21a	20.35 ± 0.07a	20.33 ± 0.07a	20.33 ± 0.07a	20.23 ± 0.02a	20.19 ± 0.02a
Woven bag	20.73 ± 0.18a	20.39 ± 0.05a	20.38 ± 0.06a	20.32 ± 0.07a	20.23 ± 0.01a	20.14 ± 0.06a
ANOVA	F = 50.04; df = 5/18; *p* < 0.01	F = 4060.00; df = 5/186; *p* < 0.01	F = 180.63; df = 5/90; *p* < 0.01	F = 38.88; df = 5/90; *p* < 0,01	F = 26.77; df = 5/90; *p* < 0,01	F = 10.16; df = 5/18; *p* < 0.01

Means in the same column followed by the same letter are not significantly different (LSD, 5%).

**Table 2:** Live insect populations (numbers of adults per 500 g of grain), damaged grains (%) and grains with eggs (%) of naturally-infested cowpea stored in hermetic and non-hermetic bags for eight months in Maradi, Niger.

Treatments	*n*	Adult Insects/500 g of Grains	*n*	% Grains with Holes	% Grains with Eggs
Initial	72	22.35 ± 0.72c	216	35.58 ± 0.51a	33.67 ± 0.71a
After 8 months					
PICS^TM^ bag	12	0.00 ± 0.00d	36	32.30 ± 1.26a	29.50 ± 1.34a
AgroZ^®^ bag	12	0.08 ± 0.08d	36	35.22 ± 1.28a	31.38 ± 1.79a
SuperGrainbag^TM^	12	0.00 ± 0.00d	36	33.11 ± 1.63a	35.03 ± 2.55a
EVAL™ bag	12	0.66 ± 0.33d	36	33.58 ± 1.38a	33.86 ± 2.41a
ZeroFly^®^ bag	12	30.33 ± 1.96b	36	97.05 ± 0.79b	97.47 ± 1.26b
Woven bag	12	39.91 ± 5.88a	36	94.66 ± 1.08b	99.47 ± 0.16b
ANOVA		F = 70.96; df = 6/137; *p* < 0.01		F = 206.68; df = 6/425; *p* < 0.01	F = 430.99; df = 6/425; *p* < 0.01

Means in the same column followed by the same letter are not significantly different (LSD, 5%).

**Table 3:** Weight of 100 grain (g) and germination (%) of naturally infested cowpea stored in hermetic and non-hermetic bags for eight months in Maradi, Niger.

Treatments	*n*	100 Grains Weight (g)	*n*	% Germination
Initial	216	14.16 ± 0.05a	144	76.41 ± 1.26a
After 8 months				
PICS^TM^ bag	36	13.73 ± 0.10a	24	60.33 ± 4.34b
AgroZ^®^ bag	36	13.77 ± 0.13a	24	61.16 ± 3.17b
SuperGrainbag^TM^	36	13.97 ± 0.13a	24	64.25 ± 3.45b
EVAL™ bag	36	13.69 ± 0.10a	24	66.50 ± 4.30b
ZeroFly^®^ bag	36	10.30 ± 0.18b	24	39.41 ± 4.54c
Woven bag	36	10.59 ± 0.15b	24	47.66 ± 4.45c
ANOVA		F = 102.71; df = 6/425; *p* < 0.01		F = 22.39; df = 6/281; *p* < 0.01

Means in the same column followed by the same letter are not significantly different (LSD, 5%).

**Table 4:** Number of abrasions on liners of hermetic bags containing naturally infested cowpea stored for 8 months in Maradi, Niger.

Treatments	*n*	Abrasions	Perforations
PICS^TM^ bag inner	4	4.00 ± 0.91a	4.00 ± 1.78 a
PICS^TM^ bag middle	4	1.25 ± 1.25a	0.00 ± 0.00 a
AgroZ^®^ bag	4	3.00 ± 1.22a	2.00 ± 1.68 a
SuperGrainbag^TM^	4	1.50 ± 0.86a	0.25 ± 0.25 a
EVAL™ bag	4	1.75 ± 1.03a	1.00 ± 0.71 a
ANOVA		F = 0.496; *df* = 3/12; *p* = 0.692	F = 0.951; *df* = 4/15; *p* = 0.447

Means in the same column followed by the same letter are not significantly different (LSD, 5%).
